# Temperament, Context and Sexual Risk among College Students

**DOI:** 10.1155/2011/504571

**Published:** 2011-06-16

**Authors:** Victoria von Sadovszky

**Affiliations:** College of Nursing, The Ohio State University, 1585 Neil Avenue, 354 Newton Hall, Columbus, OH 43210, USA

## Abstract

Much is known about predictors of risky sexual behaviors in young adults. Little is known; however, about the contribution of temperament and how temperament interacts with context to influence sexual risk intentions and actual behaviors. Since intentions are closely linked to behavior, knowing how temperament influences these decisions is important in planning interventions. The purpose of this quasiexperimental study was to examine the effect of gender, temperament, and context on sexual risk intentions and behaviors among college students (*N* = 145). Although individual components of temperament were associated with sexual risk intentions, temperament did not predict sexual risk intentions in a safer or risky context or actual behaviors. There were also no differences by gender. In this study, temperament did not interact with context to influence sexual risk intentions or behaviors. According to these results, interventions promoting safer sexual behaviors may not have to be tailored to individuals with different temperament styles.

## 1. Introduction

In the United States, sexually transmitted infections (STIs) are a major public health concern. There are an estimated 19 million new cases annually which costs the U.S. health care system $16.4 billion each year [1]. Adolescents and young adults are the most common groups to become infected and bear over half of all reported cases [1]. More concerning is the physical and psychological effects of these infections. Although most infections, even if untreated, will have no physical sequelae, those that do can lead to disorders such as pelvic inflammatory disease, sterility, and cancer [2]. The psychological effects are just as devastating. Having an STI is predictive of having higher levels of psychological distress [3] and emotional responses such as fear, denial, confusion, sadness, and anger [4]. These emotions can also inhibit disclosure of viral STIs (Herpes and Human Papillomavirus) to new sexual partners [5]. STIs are contracted through a sexual risk behaviors, and much is known about these behaviors. 

STIs are transmitted through a cluster of sexual risk behaviors (i.e., early age of intercourse, multiple sexual partners, having penetrative intercourse without a condom) and multiple correlates of these behaviors are known [6, 7]. Many studies have examined correlates and reasons for sexual risk behaviors in adolescents and young adults. Behaviors such as alcohol and drug use, carrying a weapon, and recent fighting incidents are all associated with increased sexual activity [8] and early initiation of intercourse and multiple sexual partners [7]. Perceptions such as that one's peers engage in sexual risk behaviors [9] and that one is not susceptible to STIs [10] or a lowered perceived likelihood of getting an STI [7] are positively associated with sexual risk behaviors. Once an individual has acquired an STI, there is a greater likelihood for subsequent infections [7, 10]; therefore, safer sexual behaviors, such as condom use, are paramount. Reasons why condoms are not used have been explored, and emotion is one factor. In descriptive work, participants reported being “swept away” or having sexual desire as a reason for not having safer sex [11, 12]. The likelihood or propensity of being overcome with emotions in different situations can be attributed to one's temperament [13]. Temperament has been cited as a predictor of other health risk behaviors such delinquency, substance use, and smoking [14, 15]; therefore, it stands to reason that it would be linked to sexual risk behaviors as well.

Temperament is defined as individual differences in reactivity (i.e., arousal and affect) and self-regulation(i.e., attention) [16]. These differences are influenced by heredity, maturation, and experience [17]. There is also evidence of the interaction of environment or context with temperament in determining behavior. When there is a “goodness of fit” with the environment or context, then there is optimal functioning of the organism [18, 19]. In other words, when the environmental demands are in concordance with the individual's capacities by temperamental traits, the individual is more likely to have successful behavioral outcomes. Therefore, in regards to sexual risk behaviors, it is not only important to study temperament, but also the contribution of temperament and environment to sexual risk behaviors. 

Temperament intuitively makes sense in which to examine sexual risk behaviors because the major traits of it center on emotional reactivity (i.e., arousal and affect) and self-regulation of that reactivity (i.e., attention) [20], components which are involved in sexual response. Although temperament has been studied in relation to sexual risk behaviors, only specific traits of arousal and affect primarily have been studied. Sensation seeking [21–23] and negative mood [23, 24] are related to higher sexual risk intentions (i.e., choosing risky partners, intentions not to use condoms). Novelty-seeking, harm-avoidance, reward-dependence, and avoidant coping styles are related to actual behaviors of unprotected sex [25]. Gulletteand Lyons studied sexual sensation seeking and compulsivity in relation to HIV risk behaviors and found that although men scored higher than women on sexual sensation seeking, there were no gender differences in regards to sexual risk behaviors [6]. They also found that sexual sensation seeking in conjunction with other variables (age, alcohol problems, and sexual compulsivity) predicted sexual risk behaviors. Self-regulation components of temperament, conversely, have been associated with safer sexual behaviors such as positive perceptions of sexual abstinence [26] and fewer sexual partners [27]. No studies to date have examined all temperament components (arousal, affect, and self-regulation)within one study or examined the interaction of temperament with an environmental or contextual component.

The context of a sexual encounter is made up of situational or emotional factors which influence decisions at the critical moment of deciding to engage in safer- or risky-sexual behaviors [28, 29]. Situational factors include place and partner. Aspects of the place that influence the likelihood of a sexual encounter are amount of privacy [28, 29], cozy appearance, and general comfort [28]. Partner characteristics include attractiveness of the partner [28], familiarity [12, 28] and the pressure to satisfy a partner [30]. Emotional factors can also influence the context of a sexual encounter. Individuals often report a range of emotions in sexual encounters from love to apathy [29]. It is important to note that emotions differ from mood, or the affective component of temperament in that emotions are brief and temporary experiences; whereas, temperament serves to modulate an individual's reactivity to the emotions experienced [31]. Sexual risk behaviors have been found to differ depending upon the sexual context [28]. How context interacts with temperament and affects sexual risk behaviors is unknown.

The purpose of this study was to examine all components of temperament (arousal, affect, and attention) as a predictor of sexual risk intentions in two different sexual contexts (safer and risky) and its relationship to actual sexual risk behaviors. Since temperament was different for gender in previous study [6], its association with temperament, context, and sexual risk intentions and behaviors was also examined here. It was hypothesized that sexual risk intentions would be different by context and temperament. No hypothesized directions were made since this was a first examination of temperament and context. 

## 2. Methods

### 2.1. Participants and Setting

Participants were a convenience sample of 145 college students who met the following inclusion criteria: (a) ages 18–20 and (b) able to read and write in English. A power analysis to determine sample size was conducted using Cohen's standards for multiple regression [32]. For an alpha level of  .05, a power of  .80, and a moderate effect size (.30), the desired sample size was 133 for regression analyses. A moderate effect size was selected based on the strength of the literature linking temperament, context, and sexual behavior. 

The sample was primarily female (76.3%). Participants identified themselves as either Caucasian (71.1%), African American (15.1%), Asian American (9.9%), and 3% “other.” Over half of the participants identified as being “single” (52.6%) and 45.5% were “in a relationship.” In regards to sexual health variables, 81.6% of the sample had participated in oral sex with an average age of initiation of 16.9 (SD = 1.6), 75% had experienced vaginal sex with an average age of initiation of 16.9 (SD = 1.9), and 23% had experienced anal sex with an average age of initiation of 18.5 (SD = 1.5).

### 2.2. Ethical Considerations

Prior to recruiting any participants for this study, the study was reviewed and approved by the author's University Institutional Review Board. All participants were given an informed consent with full disclosure as to the purposes, benefits, and risks involved through participation in the study. 

### 2.3. Procedure

The design of this study was quasiexperimental. The manipulated variable was context of a sexual encounter, defined as either safer or risky, based on previous research [28]. Other independent variables were temperament and gender. The dependent variables were sexual risk intentions within a context and actual sexual risk behaviors. 

Participants were recruited by placing advertisements in a local campus newspaper. Interested individuals contacted the principal investigator (PI) for information about the study and to arrange a meeting at the participant's convenience. All individuals who contacted the PI signed up to participate and completed the questionnaires. Individuals reported to a private office. The purposes of the study were explained, and participants read and signed an informed consent form. Participants first filled out a demographic questionnaire. After completing this form, participants completed the temperament scales. Finally individuals read the safer and risky scenarios. After each scenario participants checked which sexual activities (sexual intentions) they were most likely to participate in for each scenario. The scenarios were counterbalanced in order of their presentation among the participants. Participants were told not to and did not put any identifying information on any study questionnaires. Upon the completion of all the questionnaires, participants sealed all study materials in an envelope and deposited them in a box on their way out of the room. Participants received $12 for their time.

### 2.4. Measures 

#### 2.4.1. Temperament

The temperament scales are self-report instruments measuring the various components of reactivity and self-regulation of temperament as described by Derryberry and Rothbart [33, Table 1]. These scales were specifically designed for and tested on late adolescents and young adults [33]. There are a total of 180 items which comprise 18 scales that measure 6 temperament components in the major three constructs of arousal, affect, and attention. Participants answered items for each of the scales on a scale of 1, extremely untrue of me, to 7, extremely true of me. After reverse coding certain scales, scores across the scales were summed to produce a single score for each of the major temperament constructs of arousal, affect, and attention in which they were associated (Table 1). Higher scores indicated more arousal, higher negative affect, and higher levels of attentional control. With a sample of college students, the individual scales had a reliability range of  .59–  .81, with the majority of the scales higher than  .70 [33]. Reliability for the various temperament scales in this sample was also examined. Cronbach alphas were computed and ranged from  .55 to  .83 and are presented in Table 1. 

#### 2.4.2. Context and Risky Sexual Intentions Scores

Context was manipulated in this study using scenarios developed from previous work [28]. Each scenario contained key features of a safer sexual encounter (occurring in a dorm room, unattractive features of the sexual partner, partner being an acquaintance or one-time partner, and partner having good interpersonal skills) or a risky sexual encounter (partner being a boy- or girlfriend, having the encounter in a warm or cozy environment, the participant asking for the encounter, the participant finding the sexual partner attractive, the encounter after a celebration) [28]. Following each context was a question, “If you were in the situation you just read, which of the following sexual activities would you be likely to engage in?” The sexual activities from which to choose were scored as follows: abstinence (0), kissing (1), touching genitals (2), oral sex with a condom (3), vaginal sex with a condom (4), anal sex with a condom (5), oral sex without a condom (6), vaginal sex without a condom (7), to anal sex without a condom (8). Scores were summed for both scenarios giving one sexual risk intentions score for the safer context and one for the risky context. The possible range of scores was 0–36, with higher scores indicating more risky sexual intentions. A median split was performed on the scores from the safer context and the risky context. Scores above the median were considered risky and those below were considered safer.

Post-hoc tests were completed to examine whether the contexts did engender the predicted safer or risky sexual intentions. A paired *t*-test was performed on participants' sexual risk intentions scores to see if there were differences between their choices of behaviors for the safer and risky contexts. The average sexual risk intentions scores for the risky context and safer context were 10.5 (SD = 5.2) and 3.5 (SD = 3.1), consecutively. The result of the paired t test was *t*  (151) = 19.5, *P* = .001 indicating that there were significant differences in individuals' sexual risk intentions scores between the two contexts. 

#### 2.4.3. Actual Sexual Risk Behaviors

Actual sexual risk behaviors were single-item self-reports of age of initiation of intercourse and total number of vaginal, oral, and anal sex partners. These variables are strongly supported by the literature as relevant to sexual risk [7]. A median split was performed on age of first oral and vaginal sex and number of oral and vaginal partners to include in the logistic regression. 

### 2.5. Data Management and Analysis

Data were analyzed with *Statistical Package for the Social Sciences (SPSS) for Windows 17.0*. The temperament scales were examined for violations of assumptions and were found to be normally distributed and demonstrated homogeneity of variance when required. The scales also were examined for multicollinearity, and no intercorrelations were found above the .80 range. The intercorrelations on these scales ranged from *r* = −.54, *P* = .00 to *r* = .47, *P* = .00.

Bivariate Pearson correlations were used to examine each construct of temperament with the sexual risk intention scores for the safer and risky contexts and actual sexual risk behavior scores. To examine some differences between female and male participants, *t*-tests were employed. A logistic regression was also used to examine gender and temperament as a predictor of sexual intentions in both the safer and risky contexts and with actual sexual risk variables (age of first sex and number of partners for oral and vaginal sex).

## 3. Results

### 3.1. Relationships between Temperament and Risky Sexual Intentions in a Context

Table 2 shows the number of participants used in each analysis and the means and standard deviations on the major study variables.  Table 3 shows the Bivariate correlations among the major study variables. There was only one correlation between temperament and sexual risk context that was significant. Affect was positively correlated with sexual risk intentions in the safer context (*r* = .19, *P* < .05), indicating that those higher in negative affect had higher sexual risk intentions in the safer context. 

### 3.2. Relationships between Temperament and Actual Sexual Risk Behaviors

The major temperament constructs were also examined in relation to actual risk behaviors. Only one temperament construct was significantly associated with a risk behavior. Attention was negatively correlated with age of first oral sex (*r* = −.29, *P* < .01). Individuals higher in attention (maintain attention and shifting attention when desired) were more likely to engage in oral sex at earlier ages.

### 3.3. Gender Differences among the Study Variables

Gender was also examined in relation to the major study variables. Male participants had significantly higher risky sexual intentions scores in both a risky context (*t*  (152) = 2.5, *P* = .01) and a safer context (*t*  (152) = 7.4, *P* = .00) than female participants (Table 1). Conversely, female participants had higher levels of arousal than men (*t*  (139) = 2.6, *P* = .01). These relationships were also reflected in significant bivariate correlations. There were no significant associations between gender and actual sexual risk behaviors.

### 3.4. Gender and Temperament as a Predictor of Risky Sexual Intentions in Various Contexts

To determine whether gender and temperament could estimate the probability of participants making risky sexual intentions in the safer and risky context, a logistic regression was used. Seven predictor variables (Table 4) were used in the analysis. The first model ran was to predict intentions in the risky context. All independent variables were entered in one step. The Hosmer and Lemeshow test was not significant (*χ* ^2^(8, *N* = 135) = 1.49, *P* = .99) indicating that the model was a good fit. None of the variables significantly predicted risky sexual intentions. The same independent variables were entered into an equation for intentions in a safer context. The fit of this model, using the Hosmer and Lemeshow Test, was good (*χ* ^2^(8, *N* = 135) = 5.87, *P* = .66). For the safer context, like the risky context, gender and temperament were not predictive of sexual risk intentions.

### 3.5. Gender and Temperament as a Predictor of Actual Sexual Risk Behaviors

Gender and temperament were also examined in relation to age of first oral and vaginal sex and number of oral and vaginal partners (Table 4). The first model examined temperament and its interaction with gender on initiation age of vaginal sex. According to the Hosmer and Lemeshow test (*χ* ^2^(8, *N* = 135) = 8.48, *P* = .39), the model was a good fit. There were no significant findings; hence, gender and temperament were not predictive of an earlier age of vaginal debut. This was true for number of vaginal sex partners as well. Temperament and gender were not predictive of higher numbers of vaginal sex partners.

## 4. Discussion

The purpose of this study was to examine temperament and context and their contribution to sexual risk intentions and actual sexual risk behaviors. In this sample, temperament did not predict sexual risk intentions in either the safer or the risky sexual context. Temperament also did not predict actual sexual risk behaviors as well. While other studies have found that various constructs of temperament are predictive or associated with risk behaviors [21, 23, 25, 26], none have examined all three constructs together that make up arousal, affect, and attention in one study; nor, has it been looked at with the interaction of context. Thus, in this sample temperament as a whole is not a factor in risky sexual intentions or behaviors. This is a significant finding as temperamental components may not act in isolation, but more holistically to produce both risky and protective effects on intentions and behaviors.

Gender differences were also examined as this has been a significant factor in past literature in regards to certain components temperament [6]. Gender differences were examined for the temperament constructs and sexual risk intention scores. The only gender difference in temperament was related to arousal, where women were higher on that attribute than men, unlike Gullette and Lyons work where men were higher on sensation seeking [6]. There were also significant differences in how women and men rated their intentions in different sexual risk contexts. Men were more likely to choose riskier sexual behaviors on the intentions scale than women in both the safer and risky sexual contexts. In regards to actual sexual risk behaviors, however, there were no differences, similar to Gullete and Lyons [6]. It is not clear in regards to intentions related to actual behavior if women are underestimating their risks or if men are overestimating their risks in the contexts. What is known from these findings is that there were no significant differences by gender for temperament as a predictor of risky sexual intentions within the context or actual sexual risk behaviors; hence, both genders equally participated in sexual risk behaviors and temperament was not a factor. 

## 5. Limitations

This study must be examined in light of its limitations. The study was cross-sectional; therefore, behaviors over time could not be explained. The sample is homogenous, primarily female, Caucasian, and college students; hence, findings may not be representative of the population at large. The measurement of temperament was not ideal given the low internal consistency of many of the scales; thus, there may be a high amount of measurement error. It should be noted that at the time of the study, these were the best measures of temperament for young adults. Differences between these reliability findings and Derryberry and Rothbart's [33] may be explained by generational differences, further investigation is warranted. Another limitation was appropriate power for the sum of the analyses. Anal sex was not examined as the power for these analyses was only  .55 and there was a greater risk of a type II error.

## 6. Conclusions

Future research directions should include replication and longitudinal study of the interaction between temperament, context, and sexual risk behaviors with more reliable temperament scales once available. Temperament components warrant further study with respect to more specific actual sexual risk behaviors such as condom use, alcohol/drug use prior to sexual activities, and sex with high-risk partners to determine if they qualify as high-risk temperament characteristics. If so, adolescents and young adults could be tested and targeted for specific prevention interventions designed around recognizing and controlling their reactivity in the face of intensive stimuli. 

Currently, for practice, this study offers hope that developing interventions may not be dependent upon a heritable trait of emotion regulation. It would be challenging to develop interventions on a trait in which an individual has limited ability to alter a predictive style of behavior. Prior to making this conclusion, however, more research needs to occur examining temperament and its relation to sexual risk taking with prospective designs and more diverse populations.

## Figures and Tables

**Table 1:** Definitions of temperament scales.

Component	Scale	*α*	Scale definition
Arousal		.76	
Central arousal	External sensitivity	.68	Perceptual awareness of mild stimulation from external environment
	Internal sensitivity	.61	Perceptual awareness of low-intensity stimulation from within body
	Low-intensity pleasures	.72	Pleasure-related situations low-intensity situations
	Relief	.54	Pleasure derived from the attenuation of highly arousing situations
Autonomic arousal	Autonomic reactivity	.66	Autonomic activity elicited under arousing conditions
	Falling reactivity	.74	General arousal decreases from its peak to normal intensity
	Rising reactivity	.59	General arousal rises from normal to peak intensity
Motor arousal	Behavioral inhibition	.57	Capacity to suppress impulses
	Cognitive reactivity	.65	General cognitive activity (i.e., daydreaming, problem solving)
	Motor activity	.80	Extent motor system becomes activated in nondirected movements
	Motor tension	.83	Tension experienced in muscle groups throughout the body

Affect		.72	
Positive affect	Discomfort	.55	Bad affect resulting from unpleasant stimulation (i.e., pain, irritation)
	High-intensity pleasures	.66	Pleasure from situations involving high intensity
Negative affect	Fear	.66	Unpleasant affect related to unpleasant stimuli (pain, distress, etc.)
	Frustration	.60	Unpleasant affect related to interruption of tasks or blocking of a goal
	Sadness	.65	Lowered mood related to exposure of suffering, loss, and disappointment

Attention		.66	
Self-regulation	Attentional focusing	.51	Capacity to intentionally hold attentional focus on desired channels
	Attentional shifting	.51	Capacity to intentionally shift attention to desired channels

**Table 2:** Mean and standard deviations for major study variables.

	*N*	*M* (SD)	Women *M* (SD)	Men *M* (SD)
Temperament constructs				
Arousal	141	260.3 (25.0)	263.2 (24.5)	250.4 (24.6)*
Affect	145	141.8 (19.2)	142.2 (17.5)	140.6 (24.2)
Attention	144	141.3 (15.0)	140.5 (14.3)	143.9 (16.9)

Context risk scores				
Risky sexual context score	152	10.5 (5.2)	9.9 (4.8)	12.4 (6.0)*
Safer sexual context score	152	3.5 (3.1)	2.6 (2.0)	6.4 (4.3)*

Actual risky sexual behaviors				
Age of first vaginal sex	114	16.9 (1.8)	16.9 (1.7)	16.9 (1.9)
Total number of vaginal sex partners	114	5.6 (5.3)	5.5 (5.3)	5.9 (5.5)
Age of first oral sex	127	16.6 (2.1)	16.7 (1.4)	16.2 (3.3)
Total number of oral sex partners	127	4.8 (5.0)	4.6 (4.8)	5.4 (5.5)
Age of first anal sex	35	18.5 (1.5)	18.4 (1.2)	18.8 (2.3)
Total number of anal sex partners	35	1 (1.4)	1.4 (0.8)	1.0 (0)

Note:*P < .05.

**Table 3:** Correlation coefficients for major study variables.

	1	2	3	4	5	6	7	8	9	10	11	12
(1) Gender	—											
(2) Arousal	−.22*	—										
(3) Affect	−.04	.07	—									
(4) Attention	.10	.23**	−.56**	—								
(5) Risky sexual context	.21*	−.01	.10	.16	—							
(6) Safer sexual context	.52*	−.10	.19*	.02	.51**	—						
(7) Age of first vaginal sex	.00	.00	.08	.00	.00	−.01	—					
(8) Total number of vaginal sex partners	.03	−.03	.10	−.04	.14	.12	−.41**	—				
(9) Age of first oral sex	−.11	−.08	.10	−.29**	−.27**	−.10	.37**	−.18	—			
(10) Total number of oral sex partners	.07	.04	.11	.14	.33**	.20∗	−.16	.58**	−.51**	—		
(11) Age of first anal sex	.10	.22	.07	.07	−.03	−.32	.49**	.00	.12	−.03	—	
(12) Total number of anal sex partners	−.32	−.10	.05	−.07	.18	.23	−.19	.43*	.07	.40*	−.30	—

Note: *P < .05; **P < .01.

**Table 4:** Summary of logistic regression analysis predicting sexual risk intentions and actual risk behaviors.

		B	SE	Odds ratio	Wald statistic	*P*
Risky sexual context	Gender	−5.70	7.56	.00	.57	.45
	Arousal	−.07	.02	.99	.11	.74
	Affect	.03	.03	1.03	1.05	.31
	Attention	.01	.04	1.01	.16	.69
	Arousal × gender	.00	.02	.10	.03	.86
	Affect by gender	.01	.03	1.01	.06	.81
	Attention × gender	.04	.04	1.04	.85	.36

Safer sexual context	Gender	6.34	9.07	566.77	.49	.49
	Arousal	−.01	.02	.68	.17	.68
	Affect	.06	.03	1.06	3.38	.07
	Attention	.05	.04	1.05	1.39	.24
	Arousal × gender	.01	.03	1.01	.03	.86
	Affect by gender	−.04	.04	.96	1.20	.27
	Attention × gender	−.03	.05	.98	.28	.60

Age of first vaginal sex	Gender	15.38	8.92	478.46	2.97	.09
	Arousal	−.004	.01	.69	.16	.69
	Affect	.02	.02	1.01	1.31	.25
	Attention	.04	.02	1.04	3.94	.05
	Arousal × gender	−.01	.03	.10	.03	.86
	Affect by gender	−.03	.03	.44	.50	.44
	Attention × gender	−.08	.05	.93	2.29	.13

Number of vaginal sex partners	Gender	−3.89	8.53	.02	.21	.65
	Arousal	.00	.009	1.00	.20	.68
	Affect	.01	.02	1.01	.17	.78
	Attention	.01	.02	1.01	.78	.65
	Arousal × gender	.00	.03	1.00	.03	.87
	Affect by gender	.00	.03	1.00	.00	.99
	Attention × gender	.02	.05	.64	.22	.64

Age of first oral sex	Gender	4.61	8.67	100.56	.28	.99
	Arousal	.00	.01	1.00	.00	.12
	Affect	−.02	.02	.98	2.40	.26
	Attention	−.02	.02	.98	1.27	.60
	Arousal × gender	.01	.02	1.01	.25	.62
	Affect by gender	.00	.03	1.00	.00	.99
	Attention × gender	−.05	.05	.95	1.29	.26
Number of oral partners	Gender	−2.44	8.70	.09	.08	.63
	Arousal	.00	.01	1.00	.23	.09
	Affect	.03	.02	1.03	2.84	.30
	Attention	.02	.02	1.02	1.09	.78
	Arousal × gender	.03	.03	1.03	1.11	.29
	Affect by gender	−.04	.04	.96	1.01	.32
	Attention × gender	.01	.05	1.01	.01	.91
